# Political connections, investment opportunity sets, tax avoidance: does corporate social responsibility disclosure in Indonesia have a role?

**DOI:** 10.1016/j.heliyon.2022.e10155

**Published:** 2022-08-10

**Authors:** Amrie Firmansyah, Amardianto Arham, Resi Ariyasa Qadri, Puji Wibowo, Ferry Irawan, Nur Aisyah Kustiani, Suparna Wijaya, Arifah Fibri Andriani, Zef Arfiansyah, Lestari Kurniawati, Azas Mabrur, Agung Dinarjito, Rahayu Kusumawati, Moh. Luthfi Mahrus

**Affiliations:** Polytechnic of State Finance STAN, South Tangerang, Indonesia

**Keywords:** Political connections, Investment opportunity sets, Tax avoidance, Sustainability

## Abstract

This study aims to obtain empirical evidence of the effect of political connections and investment opportunity sets on tax avoidance. In addition, the use of corporate social responsibility in this study as a moderating variable aims to examine the implementation of sustainability by companies, which is a global issue of concern to many parties today. Corporate social responsibility has rarely been used in previous studies as a moderating variable in examining the relationships between investment opportunity sets and tax avoidance and political connections and tax avoidance. This study analyzed 42 manufacturing companies listed on the Indonesia Stock Exchange from 2014 to 2019, selected through a purposive sampling method to produce 252 observations. This study used a quantitative method with two-panel data regression models, namely the model and without moderation. The results suggest that political connections and investment opportunity sets positively affect tax avoidance. Meanwhile, corporate social responsibility disclosure can weaken the positive effect of political connections and investment opportunity sets on tax avoidance. This study indicates that the Indonesia Tax Authority should include sustainability issues in refining existing tax policies.

## 1 Introduction

The government considers taxes as tax collectors and companies as taxpayers. Revenue from taxation is needed by the government to finance the implementation of various government functions, provide public goods such as infrastructure and education, and various other things to improve people's welfare (Mankiw, 2012). The government's efforts to optimize tax revenues are constantly at odds with companies as taxpayers (Setyaningrum and Suryarini, 2016). From the company side, tax is one of the factors that are considered in the company's decision-making process (Lanis and Richardson, 2012) because the tax is one of the most significant business costs incurred by a company and has a direct impact on profitability and shareholder value (Landry et al., 2013). The tax expenses that burden the company and company owners cause the emergence of tax avoidance efforts (S. Chen et al., 2010). Tax avoidance is a series of activities to reduce taxes (Comprix et al., 2016; Hanlon and Heitzman, 2010; Huang et al., 2017; Puspita and Harto, 2014; Richardson, 2006). The definition of tax avoidance is also often associated with using weaknesses or loopholes in taxation provisions by taxpayers to reduce the amount of tax owed (Dyreng et al., 2008; Lim, 2011; Shafer and Simmons, 2008).

Some literature adds the scope of tax avoidance. Lietz (2013) stated that tax avoidance is part of tax planning. Besides, tax avoidance can include tax aggressiveness; in other words, tax aggressiveness is one small part of tax avoidance (Lietz, 2013). Furthermore, Kovermann & Velte (2019) explained that tax avoidance could include tax savings (tax sheltering). Thus, tax aggressiveness and sheltering can be equated to or referred to as tax avoidance measures. Tax avoidance is an exciting issue not only in the political and academic debate (Huseynov et al., 2017), but the general public has also paid more attention in response to media reports about tax avoidance practices by some global companies (Kanagaretnam et al., 2016), such as tax avoidance practices by Apple, Facebook, and Starbucks (Davis et al., 2016), the Enron case (McGill and Outslay, 2004), and the Tyco case (Wilson, 2009). Some of these cases have given the impression that tax avoidance is a common phenomenon in today's business world. The tax avoidance phenomenon was also revealed in 2016 with an investigative document of the Panama Papers. The document was processed by the International Consortium of Investigative Journalists, which contained 11.5 million investigations from 214,000 multinational companies, including shareholders and company directors, revealing the involvement of prominent world figures in offshore companies' businesses to avoid tax (Sudiarta, 2016).

The increasing difficulty of detecting or preventing tax evasion has prompted tax authorities in the Pacific Region (Australia, China, Thailand, Malaysia, and Indonesia) to discuss tax avoidance prevention in the 2018 5th Asian Tax Symposium (Direktorat Jenderal Pajak, 2018). These activities resulted in an agreement to prevent tax avoidance practices that cause the loss of potential tax revenue in a country and impact the taxation system's credibility (Direktorat Jenderal Pajak, 2018). Due to companies' tax avoidance, annual global income losses reached US $ 500 billion, with the most considerable losses occurring in low-income and middle-income countries (Cobham and Janský, 2018). Indonesia, as a middle-income country, ranks 11 out of 30 countries in terms of levels of tax avoidance as published in the databases of the International Center for Policy and Research (ICPR) and the International Center for Taxation and Development (ICTD) with the amount of tax not paid to the country being estimated at the US $ 6.48 billion per year (Cobham and Janský, 2018). Indonesia also participates in automatic information exchange cooperation agreed upon by more than 100 countries to reduce cross-border tax avoidance by companies (Hariani, 2019).

Based on the publication of Revenue Statistics in Asian and Pacific Economies (2019) - Indonesia published by the Organization for Economic Co-operation and Development (OECD), in 2017, Indonesia's tax ratio was only 11.7%, far below the average of OECD member countries (34.2%), countries in the Latin American and Caribbean Region (22.8%), and countries in Africa (18.2%) (OECD, 2020). In addition, from 2007 to 2017, Indonesia's tax ratio decreased by 0.7%, from 12.2% to 11.5% (OECD, 2020). Indonesia's tax ratio in 2019 also decreased by 0.8% compared to the previous period to 10.7% (CNN Indonesia, 2020). Based on data from the Central Government Financial Statements from 2015 to 2018, the realization of Indonesia's tax revenues did not reach the target with the respective details (CNBC Indonesia, 2019). Besides, from 2016 to 2017, the Indonesia Tax Authority has provided tax amnesty to taxpayers. As many as 956,793 taxpayers have utilized this opportunity and it generated compensation of up to Rp129 trillion (78.18%) of Rp165 trillion (Saeroji, 2017). Many parties are still trying to avoid taxes (Gloria, 2018). Data regarding the losses due to tax avoidance, high levels of tax avoidance, low tax ratios, the realization of tax revenues that did not reach the target, and the high level of utilization of tax amnesty programs in Indonesia indicate that tax avoidance in Indonesia is still a problem that needs to be investigated.

There are several suspected cases of tax avoidance practices in Indonesia. The first case is the alleged tax avoidance committed by PT Adaro Energy Tbk, which originated from a report by the international non-governmental organization Global Witness on the finding of potential tax payments that were lower than the supposed amount of US $ 125 million and tax deductions through intermediary state tax havens of US $ 14 million annually (Friana, 2018). The second case is that there are allegations of tax avoidance practices carried out by Google regarding the transfer of income earned in Indonesia to Singapore by exploiting Indonesia and Singapore (Jefriando, 2016). The tax treaty regulation states that representative companies are not included in the permanent establishment category, making it difficult for the Indonesian tax authorities to collect taxes on Google's income in Indonesia (Jefriando, 2016). These various cases encourage the need to identify factors that indicate tax avoidance actions taken by companies, especially in countries where taxes support government revenues, such as Indonesia (Tandean and Winnie, 2016).

Based on the results of a literature study that has been conducted and by referring to mapping results of Wang et al. (2019), many previous studies examined the factors influencing corporate tax avoidance activities in an international context. These studies include reviewing company size (Lisowsky, 2010), business strategy (Higgins et al., 2014), multinationality (Hope et al., 2013), institutional ownership (Khan et al., 2017), executive compensation plans (Gaertner, 2014), corporate governance (Bauer, 2016), tax enforcement (Kubick and Lockhart, 2016), external governance (Tian et al., 2016), board ties (Brown and Drake, 2014), political connections (Adhikari et al., 2006; Ajili and Khlif, 2020; Kim and Zhang, 2016; Wahab et al., 2017), set of opportunities investment (McGuire et al., 2014), and corporate social responsibility disclosure (Lanis and Richardson, 2012; Lin et al., 2017).

Meanwhile, in the Indonesian context, based on the literature review that has been carried out and refers to the mapping results of Arham et al. (2020) and Herawati et al. (2019), studies on the factors that influence tax avoidance activities carried out by companies in Indonesia has also been widely carried out. These studies include reviewing company size (Fitria and Handayani, 2019), institutional ownership (Sari and Devi, 2018), leverage (Hidayat, 2018), corporate governance (Gunawan, 2017), executive characteristics (Carolina et al., 2014), managerial skills (Nurfauzi and Firmansyah, 2018), multinational (Damayanti and Prastiwi, 2017), profitability (Saputra et al., 2015), sales growth (Turyatini, 2017), liquidity (Tiaras and Wijaya, 2015), the political connections owned by the company (Ferdiawan and Firmansyah, 2017; Iswari et al., 2019; Lestari et al., 2019; Purwanti and Sugiyarti, 2017; Sudibyo and Jianfu, 2016; Wicaksono, 2017), sets of investment opportunities they have as companies (Firmansyah and Bayuaji, 2019; Handayani, 2013; Lubis et al., 2015), as well as disclosure of corporate social responsibility (Adiputra et al., 2019; Dharma and Noviari, 2017; Fitri and Munandar, 2018; Khairunisa et al., 2017; Mulyani et al., 2017; Wijayanti et al., 2016).

This study examined the effect of political connections and investment opportunity sets on tax avoidance. Based on the political cost hypothesis in positive accounting theory, political connections and investment opportunity sets can be linked in the formulation of company policies that tend to have a motive to reduce the number of reported earnings to avoid taxes (Godfrey et al., 2010; Scott, 2015; Watts and Zimmerman, 1990). The tax avoidance activities in a company are closely related to parties' policies with interest in the company, including parties with political connections. Positive accounting theory can also explain companies' investment policy choices, as seen in investment opportunity sets. Besides, political connections are often associated with an investment; as stated by Fisman (2001), political connections are a determinant of profitability that can cause distortions in investment decisions or policies. In addition, signals from certain policies implemented by managers can recognize tax avoidance activities. Political connection and investment opportunity set are policies that can capture tax avoidance activities. These two activities can signal shareholders, creditors, employees, suppliers, and communities as company stakeholders (Canh et al., 2019).

Political connections play an essential role in many of the largest and most important economic sectors globally (Fisman, 2001) and influence companies' strategies (Goldman et al., 2009). Several academic studies have provided evidence regarding the benefits of political connections that incorporate business activities, including the size of the opportunity to get a loan (Firth et al., 2009), the imposition of favorable taxes (Faccio, 2010), the existence of government subsidies (X. Chen et al., 2008), the lack of market pressure related to public transparency (Kim and Zhang, 2016), a low probability level of tax audits and reduced tax sanctions (Li et al., 2008). Various phenomena related to companies' political connections are still not fully understood and need further investigation (Cumming et al., 2014).

In Indonesia, politicians who can create political connections in a company do not yet have a legal protection in a law (Sukmana, 2018). The existing regulations are only in the Indonesia Financial Services Authority Regulation Number 12 of 2017, which provides special treatment to politically exposed people (PEP) to prevent money laundering in the financial services sector (Sukmana, 2018). PEP in financial services sector companies based on these regulations is foreign PEPs, domestic PEPs, and people authorized by international organizations, including heads of state or heads of government, senior politicians, senior government officials, military or legal officials, senior executives at state-owned companies and political party officials (Otoritas Jasa Keuangan, 2017a). However, this study does not analyze money laundering practices in financial service sector companies but analyzes tax avoidance practices in manufacturing sector companies on the Indonesia Stock Exchange (BEI). Therefore, this study refers to Adhikari et al. (2006), Faccio (2010), Iswari et al. (2019), and Sudibyo and Jianfu (2016), which stated that a company owns a political connection if the shareholder with minimum ownership of 10% or one of the company's directors/commissioners is a member or former member of parliament, minister/cabinet member or former minister/cabinet member, member or former member of a political party, or officials or former officials of the central/regional government, including military officials.

Based on the literature review conducted, research that discusses the association between political connections and tax avoidance has been widely carried out both at the international level and in Indonesia. In the international context, the positive effect of political connections on corporate tax avoidance was found by Adhikari et al. (2006), Ajili and Khlif (2020), Kim and Zhang (2016), and Wahab et al. (2017). Meanwhile, several studies have shown that political connections positively influence corporate tax avoidance practices (Ferdiawan and Firmansyah, 2017; Sudibyo and Jianfu, 2016). Meanwhile, the negative effect of political connections on corporate tax avoidance activities has been found (Iswari et al., 2019; Wicaksono, 2017). On the other hand, Lestari et al. (2019) and Purwanti and Sugiyarti (2017) suggested that political connections do not affect tax avoidance. The different results of studies conducted both outside and in Indonesia still suggest inconsistencies, which means that further investigation is still needed.

The investment opportunity set resulting from the company's investment policy can show the company's growth rate and is responded to positively by the market, which leads to an increase in investment in the company (Vogt, 1997). In line with this, Smith and Watt (1992) also found opportunities for companies to continue to grow through investment opportunities seen from various combinations of investment opportunity sets. The investment opportunity set combines tangible assets owned by the company and profitable investment choices (Myers, 1977). In Indonesia, the provisions regarding investment opportunity sets have not been expressly regulated in statutory regulations or accounting standards. However, investment activities carried out by companies are regulated in Indonesia’s Law Number 25 of 2007 concerning Investment. Based on a report from the Investment Coordinating Board, investment realization of Indonesian companies from 2014 to 2019 showed a positive trend or has increased in detail per year, amounting to IDR 156.1 trillion in 2014, IDR 179.4 trillion in 2015, IDR 216.3 trillion in 2016, IDR 262.3 trillion in 2017, IDR 328.6 trillion in 2018, and IDR 386.5 trillion in 2019 (BKPM, 2020). The investment opportunity set is also one factor that companies consider in formulating business strategies, including tax planning strategies, which include tax avoidance practices (McGuire et al., 2014). The various essential roles of investment opportunity sets and their relationship with a company's tax avoidance encourage this study to examine the effect of investment opportunity sets on tax avoidance practices carried out by companies.

Previous studies linking the investment opportunity set with tax avoidance are still limited in number. Previous studies have generally reviewed the effect of investment opportunity sets on firm value (Fauzy et al., 2019; Kolibu et al., 2020; Resti et al., 2019), company dividend policy (Abor and Bokpin, 2010; Chintya et al., 2018; Mutiarahim, 2019), as well as the earnings quality (Hutagalung et al., 2018; Putra and Subowo, 2016). Only one study discusses the effect of the investment opportunity set on tax avoidance in an international context, namely McGuire et al. (2014). The study examined the effect of investment opportunity sets on tax shelters' investment activities as a form of tax avoidance. This study indicates that the investment opportunity set has a negative effect on tax avoidance. Meanwhile, in Indonesia, studies that examined the effect of investment opportunity sets on tax avoidance have been conducted by Firmansyah and Bayuaji (2019), Handayani (2013), and Rusli et al. (2015). Firmansyah and Bayuaji (2019) and Rusli et al. (2015) and found a negative effect of tax avoidance's investment opportunities. However, Handayani (2013) provided empirical evidence that the investment opportunity set does not affect tax avoidance. The results of studies of the effect of the investment opportunity set on tax avoidance, which still show inconsistencies, encourage this study to investigate further. Besides, in terms of investment realization, from 2014 to 2019, one of the sectors has always been included in the category of the two largest sectors in the manufacturing sector (BKPM, 2020). Therefore, this study examines the effect of investment opportunity sets on tax avoidance in manufacturing companies listed on the Indonesia Stock Exchange.

In addition, this paper incorporated corporate social responsibility disclosure as a moderating variable, which makes this paper unique among contemporary tax avoidance works of literature. Wijaya and Arumningtias (2021) noted the presence of tax avoidance gaps relating to the execution of Indonesian government legislation that went into force in 2013 and 2018. Another paper delves into tax avoidance using emerging data as an independent variable on financial performance (Khuong et al., 2020), earnings management (Putri et al., 2016), and firm value (Irawan and Turwanto, 2020; Widodo and Firmansyah, 2021). Other studies examined tax avoidance by employing emerging data as an independent variable on financial performance (Khuong et al., 2020), earnings management (Putri et al., 2016), and firm value (Irawan and Turwanto, 2020; Widodo and Firmansyah, 2021). Muthitacharoen and Samphantharak (2020) investigated tax avoidance in profit shifting through transfer pricing activities by employing multinational firms in the ASEAN region, while Firmansyah and Yunidar (2020) employed Indonesian enterprises. Oktavia et al. (2019) conducted a tax avoidance assessment incorporating aggravating factors.

Previous studies simply presented the relationship between tax avoidance and other variables but did not explain the drivers typically observed in tax audit practice in emerging economies such as Indonesia. Furthermore, previous studies have not explored the suggested role of corporate social responsibility in obstructing the relationship between political connections and investment opportunities focused on tax avoidance. Our study analyzed four features of a country's tax environment, including the tax base, the application of income tax on dividends, the interval of fiscal losses, and the appropriateness of tax planning, to fill in the gaps in prior studies. This study employed corporate social responsibility disclosure as an aspect of corporate moral obligation in moderating the impact of political connections and the investment opportunity set owned by Indonesian corporations on tax avoidance behaviors. The positioning of corporate social responsibility is critical in determining if tax avoidance is linked to the global sustainability response, which is now in growth. Regulators in Indonesia, however, primarily control environmental components of corporate social responsibility, although, in actuality, it encompasses economic, environmental, and social aspects (Firmansyah and Estutik, 2020; Gunawan, 2017).

Based on stakeholder theory, a company is responsible to its owners or shareholders and other parties interested in it (Freeman, 2010), which affects its survival (Mitchell et al., 1997). Corporate social responsibility disclosure is a medium for company communication with stakeholders (McWilliams and Siegel, 2001) because companies have a moral obligation to consider and align all stakeholders' interests (Freeman, 2010). By the stakeholder theory, companies that carry out corporate social responsibility activities as a form of moral responsibility and accountability to society also do the same to the government through proper tax obligations. Implementing corporate social responsibility activities can align the community and the government's interests as the company's stakeholders. Taxes also closely relate to corporate social responsibility because they can benefit the community (Avi-Yonah, 2008). Taxes are part of the company's social responsibility (Fallan and Fallan, 2019). In Indonesia, to date, no law explicitly regulates corporate social responsibility. However, the drafting and deliberation of the CSR bill have been a priority national legislation program for the House of Representatives since 2017 (Kliklegal.com, 2017). It is due to the pros and cons of the law, especially among business actors (Kontan.co.id, 2018).

However, there are several laws and regulations that regulate the implementation of corporate social responsibility activities (Kliklegal.com, 2017). The first law is Law Number 40 of 2007 concerning Limited Liability Companies that regulates the obligations of companies whose business activity related to natural resources to carry out social and environmental responsibility activities. The second is Government Regulation Number 47 of 2012 concerning Social and Environmental Responsibility of Limited Liability Companies that regulates the mechanism for implementing corporate social and environmental responsibility. The implementation of corporate social responsibility can be identified through the publication or disclosure of information regarding the implementation of corporate social responsibility activities. It has been the research object globally in developed and developing countries since the early 1980s (Ali et al., 2017). In Indonesia, up to 2020, laws and regulations and accounting standards have not obligated all public companies to present and disclose information about the implementation of corporate social responsibility activities. However, companies have disclosed this information in their annual or sustainability reports.

Several studies have reviewed the effect of corporate social responsibility disclosure on tax avoidance. Lanis and Richardson (2014) found a negative effect of corporate social responsibility disclosure on tax avoidance at an international level. Other studies found that corporate social responsibility disclosure positively affects tax avoidance (Gulzar et al., 2018; Lin et al., 2017). Meanwhile, in Indonesia, most studies showed a negative effect of disclosure of corporate social responsibility on tax avoidance (Adiputra et al., 2019; Dharma and Noviari, 2017; Fitri and Munandar, 2018; Khairunisa et al., 2017; Mulyani et al., 2017; Ratmono and Sagala, 2015). Other studies have proven that implementing corporate social responsibility activities can improve company performance because companies can maintain long-term commitments that are the concern of stakeholders (Cherian et al., 2019) and lower tax risk (Thuy et al., 2021). In addition, Khuong et al. (2022) concluded that corporate social responsibility activities can improve the financial reporting quality because companies that implement corporate social responsibility are considered capable of maintaining connections with stakeholders and fulfilling the wishes of stakeholders.

Thi et al. (2021) examined the company's financial performance using an index developed from disclosure claims made by the company. Meanwhile, the usage of corporate social responsibility disclosures in our study employed the quality of disclosure based on scores as Lee (2017), Firmansyah and Estutik (2020), and Vito et al. (2022). Therefore, this study's corporate social responsibility disclosure can suggest the quality of the implementation of corporate social responsibility in Indonesia as one of the emerging market countries.

This study also included several control variables selected based on the frequency of use and their significance level in the previous studies related to tax avoidance. There were three control variables in this study, namely, company size, leverage, and profitability. Large companies have a higher likelihood of tax avoidance (Ardianto and Rachmawati, 2018; Swingly and Sukartha, 2015; Wardani and Khoiriyah, 2018). Meanwhile, according to Lanis and Richardson (2014) and Mulyani et al. (2017), the level of corporate debt (leverage) has a positive effect on tax avoidance because debt interest payments can be used as a deduction from the tax expense. Regarding profitability, Comprix et al. (2016), Dwiyanti and Jati (2019), and Oktavia and Martani (2013) provided empirical evidence of a positive effect of profitability on tax avoidance. Thus, using company size, leverage, and profitability as control variables in this study is expected to explain the existing phenomena because it considers factors other than the independent variables being tested.

By combining the more relevant factors usually found in the tax audit sector in developing nations, particularly political connections and investment opportunity sets, this study adds empirical novelty to the growth of financial accounting research connected to tax avoidance activity. Therefore, this research may be a starting point for future studies on the relevance of corporate social responsibility disclosure in tax avoidance testing. In a more practical sense, the Indonesian Tax Authority can use the findings of this study to develop tax policies in the areas of politics, social policy, economics, and the environment. It can also help tax auditors establish whether or not a company is devoted to tax avoidance.

## 2 Methodology

This study employed secondary data from manufacturing sector companies listed on the Indonesia Stock Exchange from 2014 to 2019. The data includeed financial report data, annual reports, sustainability reports, and other relevant data obtained from www.idx.co.id and its official website. The population used in this study were all companies listed on the Indonesia Stock Exchange as of June 30, 2020. This study selected a sample using the purposive sampling method with the following criteria:

From Table 1, manufacturing companies with a negative amount of pre-tax income or experiencing losses were excluded from this study because it can cause misleading in calculating the tax expenses, resulting in distortion of the measurement of the tax avoidance variable (Hanlon and Heitzman, 2010). Negative income before tax is unable to identify the differences in tax between accounting income and fiscal income because the calculation of fiscal income employs the formula in which tax expense is divided by tax rate so that if income before tax is negative, it indicates that there are no tax payments or the value of the tax expenses is zero, which causes fiscal income is also zero. Thus, the tax difference cannot be described.

**Table 1:** Research sample.

No.	Criteria	Number
1	Companies listed on the IDX as of June 30, 2020	702
2	Companies on the IDX engaged in sectors other than manufacturing	-526
3	Manufacturing companies listed on the IDX after January 1, 2014	-55
4	Manufacturing companies that were delisting or relisting during the period 2014 s.d. 2019	-1
5	A manufacturing company with negative pre-tax income	-54
6	Manufacturing companies with a fiscal year-end other than December 31	-1
7	Manufacturing companies that have incomplete data for the period 2014–2019	-23
**Number of companies for sample**		**42**
**Year**		**6**
**Total sample**		**252**

This study's dependent variable was tax avoidance, while the independent variable was political connections and investment opportunity sets. This study used corporate social responsibility disclosure as a moderating variable and company size, leverage, and profitability as control variables. The operational definition and proxies used for each variable are as follows. Tax avoidance is a series of activities to reduce taxes (Hanlon and Heitzman, 2010; Huang et al., 2017). The definition of tax avoidance is also often associated with the utilization of weaknesses in tax provisions by taxpayers (Brian and Martani, 2016; Dyreng et al., 2008). To overcome various limitations in tax avoidance measurement, the proxy used in this study was abnormal permanent differences originating from manager discretion (DTAX). According to Frank et al. (2009), using DTAX proxies in measuring the level of tax avoidance can better detect any efforts to reduce taxable profits aimed at tax avoidance. In its development, besides referring to the model of Frank et al. (2009), the DTAX proxy has also been adjusted to the context of Indonesia by Rachmawati and Martani (2017) and it has been used by Aryotama and Firmansyah (2019), Saksessia and Firmansyah (2020) as in Eq. (1)

(1) $${\text{PERMDIFF}}_{\text{it}}={\text{α}}_{0}+{\text{α}}_{1}{\text{INTANG}}_{\text{it}}+{\text{α}}_{2}\Delta {\text{NOL}}_{\text{it}}+{\text{α}}_{3}{\text{LAGPERM}}_{\text{it}}+{\text{ε}}_{\text{it}}$$

Where:

${\text{PERMDIFF}}_{\text{it}}$ : the permanent difference between income accounting-based and income tax-based, namely the total book-tax difference minus the temporary difference in company i year t, or $\left[{\text{BI}}_{\text{it}}-\left(\frac{{\text{CTE}}_{\text{it}}}{{\text{STR}}_{\text{it}}}\right)\right]-\left(\frac{{\text{DTE}}_{\text{it}}}{{\text{STR}}_{\text{it}}}\right)$, which is scaled by total assets t-1
${\text{BI}}_{\text{it}}$ : accounting profit before tax company i in year t
${\text{CTE}}_{\text{it}}$ : the current tax expense for the company i in year t
${\text{DTE}}_{\text{it}}$ : deferred tax expense for the company i in year t
${\text{STR}}_{\text{it}}$ : statutory tax rate (corporate income tax rate under the Income Tax Law) in year t
${\text{INTANG}}_{\text{it}}$ : goodwill and other intangible assets for the company i in year t are scaled against total assets
$\Delta {\text{NOL}}_{\text{it}}$ : change in the net operating loss that can be compensated for the company i in year t, scaled to total assets t-1
${\text{LAGPERM}}_{\text{it}}$ : PERMDIFF one year earlier for the company i in year t, scaled to total assets t-1
${\text{ε}}_{\text{it}}$ : abnormal permanent differences stemming from managers' discretion for firm i in year t (DTAX)

The DTAX value in this study was obtained from the regression results in a cross-sectional manner. Cross-sectional regression was chosen because it can capture differences in tax avoidance from year to year due to changes in industrial conditions and economic policies in the year concerned (Saksessia and Firmansyah, 2020).

This study refers to political connections in Adhikari et al. (2006), Faccio (2010), Iswari et al. (2019), and Sudibyo and Jianfu (2016), which stated that a company has political connections if the shareholder who owns at least 10% of the total shares or one of the company's directors/commissioners is:

1) members or former members of parliament,
2) minister/cabinet member or former minister/cabinet member,
3) members or former members of a political party, or
4) officials or former officials of central/regional government, which includes the military forces.

To determine whether there are political connections in a company, this study examined the company shareholders who own at least 10% of total shares and examined the board of directors' and commissioners' profiles through the company's annual report. In addition, this study also investigated further by extracting information via the internet. This study employed a proxy for political connection, referring to Lin et al. (2018), to show whether or not there is a political connection and to describe the strength of the political connection the company has as presented in Eq. (2).

(2) $${\text{POLCON}}_{\text{it}}=\text{LN ​}\left(1+\text{Politically ​Connected ​Board ​Member}\right)$$

In this study, investment opportunity is the company's tangible assets or resources and the company's ability to continue to grow by investing in various profitable investment options for the company (Kallapur and Trombley, 2001; Myers, 1977). The investment opportunity set is measured using a combined proxy to minimize the default calculation error contained in a single proxy. This proxy is considered to produce a better measurement than a single proxy (Smith and Watts, 1992). The combined size-based proxies consist of five single proxies that have been widely used in previous studies (Firmansyah and Bayuaji, 2019; Gaver and Gaver, 1993; Kallapur and Trombley, 2001; McGuire et al., 2014), as in Eqs. (3), (4), (5), (6), and (7).

1) Market to book value of equity (MVEBVE)

(3) $$\text{MVEBVE}=\frac{\text{Number ​of ​Outstanding ​Shares ​X ​Closing ​Price}}{\text{Total ​Equity}}$$

2) Market to book value of assets (MVABVA)

(4) $$\text{MVABVA}=\frac{\text{Total ​Asset}-\text{Total ​Equity}+\left(\text{Outstanding ​Share ​X ​Closing ​Price}\right)}{\text{Total ​Asset}}$$

3) Earning to price ratio (EPR)

(5) $$\text{EPR}=\frac{\text{Earning ​per ​Shares}}{\text{Closing ​Price}}$$

4) The ratio of capital expenditure to book value of assets (CAPBVA)

(6) $$\text{CAPBVA}=\frac{\left({\text{Book ​Value ​of ​Fixed ​Assets}}_{\text{t}}-{\text{Book ​Value ​of ​Fixed ​Assets}}_{\text{t}-1}\right)}{\text{Total ​Asset}}$$

5) The ratio of capital expenditure to the market value of assets (CAPMVA)

(7) $$\text{CAPMVA}=\frac{\left({\text{Book ​Value ​of ​Fixed ​Assets}}_{\text{t}}-{\text{Book ​Value ​of ​Fixed ​Assets}}_{\text{t}-1}\right)}{\text{Total ​Asset}-\text{Total ​Equity}+\left(\text{Outstanding ​Share ​X ​Closing ​Price}\right)}$$

In this study, the five single proxies for the investment opportunity set were reduced through factor analysis to obtain a combined factor score representing the investment opportunity set variable. The variables used are those with significant correlations. First, the single proxy variables used for the investment opportunity set have a Kaiser Meyer Olkin (KMO) value above 0.5 and a significance level of less than 0.05 (Firmansyah and Bayuaji, 2019). Second, the correlation between the investment opportunity set variables was tested by measuring sampling adequacy (MSA). A variable must be excluded from the factor analysis process if it has an MSA value less than 0.5. Next, the variables with MSA values above 0.5 were re-tested by factor analysis (Hair et al., 2014). Third, the number of form factors is determined based on the eigenvalues. If there are factors with eigenvalues more than or equal to 1, that factor can be used because it is considered to have represented all variables (Hair et al., 2014). The last eigenvalues with a value greater than or equal to 1 are selected as the extraction stop points. Finally, the number of factors used is based on the total cumulative amount of variation achieved. Suppose that the cumulative total variation has exceeded 75%. The factor formed is considered sufficient to explain the variables of the investment opportunity set, which means that factor extraction can be stopped. Next, the factor analysis process is continued by determining the factor members utilizing factor loading. A variable can be categorized into a certain factor if it has a high factor loading value on one of the factors. The final step in the factor analysis process is forming a factor score. If more than one factor is formed, all the factors formed are added to become only a one-factor index (Firmansyah and Bayuaji, 2019). The factor score obtained through the analysis of these factors then becomes the proxy value for the investment opportunity set.

Disclosure of corporate social responsibility delivers information related to company activities that have economic, environmental, and social impacts to meet stakeholders' demands that affect the company's survival (Adams and McNicholas, 2007; Deegan, 2014). Corporate social responsibility disclosure is measured using the Global Reporting Initiatives (GRI) G4 index because it is an international best practice regarding sustainability reporting to the public, which contains positive or negative contributions of an organization to achieving sustainable development goals.

This study employed the scale listed in Table 2 in providing a score for each item of disclosure in the annual report/sustainability report through content analysis. The scale scores were summed and then compared with the number of disclosure indicators based on GRI G4 in Lee (2017) and Firmansyah and Estutik (2020) as shown in Eq. (8).

(8) $${\text{CSRD}}_{\text{it}}=\;\frac{\text{Total ​Score ​Disclosure}}{\text{Number ​of ​Disclosure ​Criteria ​Based ​on ​GRI ​G}4}$$

**Table 2:** CSRD index scale.

Scale	Description
0	Do not disclose
1	Minimum disclosure
2	Descriptive disclosure
3	Quantitative disclosure
4	Truly extraordinary

This study also included company size, leverage, and profitability as control variables because of the frequency of use and the level of significance in previous studies related to tax avoidance (Dwiyanti and Jati, 2019; Swingly and Sukartha, 2015). Firm size (SIZE) was used to control for the effect of economies of scale. SIZE was measured using the natural logarithm (ln) of the company's total assets as done by Lanis and Richardson (2012), Swingly and Sukartha (2015), Wardani and Khoiriyah (2018). Leverage (LEV) needs to be controlled because interest expense from debt is a deduction from gross income and thus, it needs to be controlled so that tax savings do not come from high debt. LEV was measured using the ratio of total long-term debt to total assets in the current year as done by Lanis and Richardson (2014) and Mulyani et al. (2017). Profitability (ROA) needs to be controlled because company performance can cause taxes to change from year to year. Profitability was measured using the profit ratio before tax divided by total assets in the current year as done by Comprix et al. (2016), Lanis and Richardson (2013), and Oktavia and Martani (2013).

Hypothesis was tested by using multiple linear regression analysis for panel data. According to Gujarati and Porter (2009), the selection of the most appropriate multiple linear regression model for panel data needs to be conducted. The Chow test is a test to compare the common effect model and the fixed effect model. The Breusch-Pagan Lagrange multiplier test is a test to compare the common effect model and the random effect model in determining the most appropriate model. The Hausman test is a test to compare the fixed effect model and the random effect model in determining which model is the most appropriate. This study employed two research models. The first model, which is in Eq. (9), is a research model used to examine political connections and investment opportunity sets on tax avoidance according to the first and second hypotheses.

(9) $${\text{DTAX}}_{\text{it}}={\text{β}}_{0}+{\text{β}}_{1}{\text{POLCON}}_{\text{it}}+{\text{β}}_{2}{\text{IOS}}_{\text{it}}+{\text{β}}_{3}{\text{SIZE}}_{\text{it}}+{\text{β}}_{4}{\text{LEV}}_{\text{it}}+{\text{β}}_{5}{\text{PROF}}_{\text{it}}+{\text{ε}}_{\text{it}}$$

The second model in Eq. (10) is the research model used to examine the role of corporate social responsibility disclosure in moderating the effect of political connections and investment opportunity sets on tax avoidance according to the third and fourth hypotheses.

(10) $${\text{DTAX}}_{\text{it}}={\text{β}}_{0}+{\text{β}}_{1}{\text{POLCON}}_{\text{it}}+{\text{β}}_{2}{\text{IOS}}_{\text{it}}+{\text{β}}_{3}{\text{CSRD}}_{\text{it}}+{\text{β}}_{4}\left({\text{POLCON}}_{\text{it}}*{\text{CSRD}}_{\text{it}}\right)+{\text{β}}_{5}\left({\text{IOS}}_{\text{it}}{\text{CSRD}}_{\text{it}}\right)+{\text{β}}_{6}{\text{SIZE}}_{\text{it}}+{\text{β}}_{7}{\text{LEV}}_{\text{it}}+{\text{β}}_{8}{\text{PROF}}_{\text{it}}+{\text{ε}}_{\text{it}}$$

${\text{DTAX}}_{\text{it}}$ : company i tax avoidance in year t
${\text{POLCON}}_{\text{it}}$ : company i's political connections in year t
${\text{IOS}}_{\text{it}}$ : set of investment opportunities for the company i in year t
${\text{CSRD}}_{\text{it}}$ : corporate social responsibility disclosure for the company i in year t
${\text{SIZE}}_{\text{it}}$ : the size of the company i in year t
${\text{LEV}}_{\text{it}}$ : leverage of company i in year t
${\text{PROF}}_{\text{it}}$ : profitability of company i in year t
${\text{ε}}_{\text{it}}$ : error
β: constant

## 3 Results

Table 3 shows the descriptive statistics for each variable employed in this study:

**Table 3:** Descriptive statistics.

Var	Obs	*Mean*	Median	*Std. Dev*	Min	Max.
DTAX	252	4.41E-19	-0.0010	0.0238	-0.0827	0.2264
POLCON	252	0.5203	0.6931	0.5569	0.0000	1.9459
IOS	252	1.98E-07	-0.3085	1.4142	-2.3204	12.2844
CSRD	252	0.4690	0.3846	0.3296	0.0000	1.8681
SIZE	252	29.1863	28.8989	1.7266	25.7957	33.4945
LEV	252	0.1368	0.1027	0.1273	0.0013	0.7217
PROF	252	0.1048	0.0822	0.0815	0.0014	0.4002

The Pearson correlation matrix was used to perform the multicollinearity test. There is a strong correlation between the independent variables if the correlation matrix between two variables in the Pearson correlation matrix displays a number greater than 0.90. It demonstrates that there is an issue with multicollinearity. The regression model has passed the multicollinearity test if the correlation matrix value between two variables is less than 0.90 (Gujarati and Porter, 2009). Table 4 shows the results of the multicollinearity test for model 1, and Table 5 shows the results for model 2. According to Tables 4 and 5, the correlation matrix between the two variables in the Pearson correlation matrix has a value of less than 0.90.

**Table 4:** Model 1 multicollinearity test result.

Variable	DTAX	POLCON	IOS	SIZE	LEV	PROF
**DTAX**	1.0000					
**POLCON**	0.1941	1.0000				
**IOS**	0.1447	0.0562	1.0000			
**SIZE**	0.1005	0.2413	0.1050	1.0000		
**LEV**	-0.0043	-0.1374	-0.1079	0.2992	1.0000	
**PROF**	0.1561	0.1705	0.3889	0.1406	-0.3119	1.0000

**Table 5:** Model 2 multicollinearity test result.

Variable	DTAX	POLCON	IOS	CSRD	POLCON∗CSRD	IOS∗CSRD	SIZE	LEV	PROF
**DTAX**	1.0000								
**POLCON**	0.1941	1.0000							
**IOS**	0.1447	0.0562	1.0000						
**CSRD**	0.0419	0,2628	0.0677	1.0000					
**POLCON∗CSRD**	0.1224	0.7433	0.0091	0.7142	1.0000				
**IOS∗CSRD**	0.0820	-0.0198	0.7843	0.1313	-0.0521	1.0000			
**SIZE**	0.1005	0.2413	0.1050	0.5147	0.4494	0.0053	1.0000		
**LEV**	-0.0043	-0.1374	-0.1079	0.0927	0.0286	-0.114	0.2992	1.0000	
**PROF**	0.1561	0.1705	0.3889	0.0344	0.0365	0.3373	0.1406	-0.3119	1.0000

In addition, the interaction variable of corporate social responsibility in Table 5 reveals that there was no issue with multicollinearity compared to other factors. Suppose no multicollinearity issues are detected when the moderator is included in the model. The interaction variable test satisfies Baron and Kenny’s (1986) criteria to include the moderating variable in the regression model. This indicates that neither model 1 nor model 2 has a problem with multicollinearity. Consequently, this study's multiple linear regression equation passed the multicollinearity test.

After performing the Chow test, Hausman test, and Lagrange Multiplier test, the best model for both model 1 and model 2 was a fixed-effect model. The resulting multiple linear regression is presented in Table 6. Model 1 is a model that aims to examine the independent variable on the dependent variable, while model 2 aims to examine the role of corporate social responsibility disclosure in moderating the association between the independent variables and the dependent variable:

**Table 6:** The result of hypothesis testing.

Variable	Sign	Model 1			Model 2		
		Coeff	t-Stat	Prob.	Coeff	t-Stat	Prob.
C		-0.164	-1.24	0.111	-0.143	-1.01	0.159
POLCON	+	0.017	2.69	0.005∗∗∗	0.029	3.12	0.002∗∗∗
IOS	+	0.003	1.83	0.037∗∗	0.005	3.28	0.001∗∗∗
SIZE		0.005	1.12	0.134	0.004	0.82	0.208
LEV		0.002	0.07	0.476	0.001	0.04	0.485
PROF		0.134	1.34	0.093∗	0.139	1.39	0.087∗
CSRD					0.017	2.19	0.018∗∗
POLCON∗CSRD	-				-0.021	-2.39	0.011∗∗
IOS∗CSRD	-				-0.005	-2.60	0.007∗∗∗
R^2^		0.103			0.118		
F-Stat.		2.46			2.33		
Prob. F		0.048			0.037		

Annotation: POLCON is political connections, IOS: investment opportunities set, SIZE: the size of the company, LEV: leverage, PROF: profitability; CSRD: corporate social responsibility disclosure. ∗, ∗∗, and ∗∗∗ denote significance based on one-tailed t-tests at or below the 10%, 5%, and 1% levels.

## 4 Discussion

### 4.1 The association of political connections and tax avoidance

Based on the results of hypothesis testing, political connections were positively associated with tax avoidance. The result of hypothesis testing in this study confirms the results of studies conducted by Adhikari et al. (2006), Ajili and Khlif (2020), Ferdiawan and Firmansyah (2017), Kim and Zhang (2016), Sudibyo and Jianfu (2016), and Wahab et al. (2017). However, this study's result is not in line with Iswari et al. (2019) and Wicaksono (2017). This study is also not in line with Lestari et al. (2019) and Purwanti and Sugiyarti (2017), which suggested the absence of the influence of political connections owned by companies on the level of the company's tendency to avoid taxes. This study confirms a positive accounting theory that explains companies' choice of accounting policies (Godfrey et al., 2010; Watts and Zimmerman, 1990). In preparing accounting policies in a company, parties interested in the company, such as directors, commissioners, and shareholders, cannot be avoided. These parties' strong political connections can affect company policies, including taxation policy. The political cost hypothesis in positive accounting theory states that company policy choices reduce tax avoidance (Scott, 2015). Therefore, choosing company policies that involve parties with political connections tends to lead to tax avoidance practices. Thus, companies with strong political connections also tend to avoid taxes.

The strength of political connections in a company is indicated by the number of directors/commissioners/shareholders who have held or are currently holding positions in the parliament, cabinet, political parties, central/regional government, and military forces. Based on the descriptive statistical analysis results, the manufacturing companies observed in this study had low political connection power. Some manufacturing companies had no political connections. However, some manufacturing companies had strong political connections. In Indonesia, no provisions regulate politicians' involvement in business, creating political connections in public companies. Based on the results of hypothesis testing in this study, if a manufacturing company has political connections, it tends to be used by the company to engage in tax avoidance activities. In the context of Indonesia, empirical evidence that has been obtained in previous research is the ability of political connections to trigger tax avoidance, which is marked by the low Cash Effective Tax Rate (ETR) owned by manufacturing companies (Ferdiawan and Firmansyah, 2017; Sudibyo and Jianfu, 2016). Cash ETR shows the ratio between the company's amount of cash for tax payments and its profit before tax (S. Chen et al., 2010). The amount of cash paid for tax and profit before tax can be known explicitly in the company's financial statements.

Meanwhile, this study measured tax avoidance based on the value of abnormal permanent differences originating from company managers' discretionary results (DTAX), which are implicitly or not expressly disclosed in the company's financial statements. Even with different tax avoidance measures, this study also provides empirical evidence that manufacturing companies in Indonesia tend to use political connections to avoid taxes. It suggests that, in Indonesia, the empirical evidence obtained provides the same results using both explicit and implicit tax avoidance measures.

In several countries, including Malaysia (Adhikari et al., 2006; Wahab et al., 2017) and Iran (Ajili and Khlif, 2020), the empirical evidence obtained also shows that companies with political connections tend to avoid taxes. The tendency of politically connected companies to avoid taxes is also found in the United States (Kim and Zhang, 2016). If it is traced further, Malaysia, Iran, and the United States adhere to the same tax system as Indonesia, the self-assessment system (Djulianto, 2015). A self-assessment system is a taxation system that gives taxpayers the confidence to calculate, pay, and report their taxes (Djulianto, 2015). The tax paid to the tax authorities depends on the profit in the financial statements. With the existence of political connections owned by companies, companies in countries that implement self-assessment systems tend to be more willing to reduce the amount of reported profit so that the amount of tax that must be paid to tax authorities is reduced (Godfrey et al., 2010; Scott, 2015). It also confirms that when a company has political connections in a country that adopts a self-assessment tax system, it implements a tax avoidance strategy when it prepares financial statements by consulting parties with political connections. Thus, companies in countries that adopt a self-assessment tax system and have political connections tend to use the opportunity to make it easier for them to avoid taxes.

### 4.2 The association of investment opportunity sets and tax avoidance

Referring to the results of hypothesis testing, the investment opportunity set is positively associated with tax avoidance. The result of this study is not in line with Firmansyah and Bayuaji (2019), McGuire et al. (2014), and Rusli et al. (2015). This study is also not in line with Handayani (2013). However, this study follows the political cost hypothesis in positive accounting theory (Godfrey et al., 2010; Watts and Zimmerman, 1990). This study confirms the political cost hypothesis in positive accounting theory, which states that companies tend to have a motive to reduce the amount of taxes paid to the government (Godfrey et al., 2010). The company executes this motive by accounting policies and practices that can benefit the company (Adam and Goyal, 2008; Kallapur and Trombley, 2001). One type of accounting policy and practice is an investment, which can be seen from its investment opportunity set.

As an accounting policy or practice that benefits the company, the investment opportunity set is closely related to decision-making to formulate business strategy. Taxes are never left as consideration for companies (Lanis and Richardson, 2012). Taxes are also the most significant business expense for companies, so the formulation of corporate investment policies tends to reduce the amount of taxes (Lanis and Richardson, 2012).

As a result of policies to reduce taxes, the investment opportunity set indicates that a company avoids taxes. Therefore, the investment opportunity is a company strategy to practice tax avoidance. This study provides empirical evidence that companies with a large set of investment opportunities also tend to exercise tax avoidance. The empirical evidence obtained in this study is the positive influence of investment opportunity sets on tax avoidance, which is also related to company growth (Adam and Goyal, 2008; Gaver and Gaver, 1993), which can produce complex information about company business transactions (Cahan et al., 2008; Smith and Watts, 1992). An increase in the number of investment opportunities can cause the company to continue to grow to become a more prominent company.

This consequence is an increase in companies' information related to business transactions. Every company's business activity, including selecting investment expenditure, involves company managers' discretion (Adam and Goyal, 2008; Gaver and Gaver, 1993). The manager's discretion is not easy to predict and monitor by external parties, including the tax authorities. A company's number of investment opportunities has impacted the number and types of business transactions reported to the tax authorities (McGuire et al., 2014).

Companies with large investment opportunities report diverse and unique business transaction information. It can be a challenge for tax authorities to conduct supervision. Tax authorities need extra knowledge, time, and effort to identify each transaction information to detect them and determine which business transactions indicate tax avoidance tendencies. In contrast, firms with a small set of investment opportunities report few and homogeneous business transactions that can make it easier for tax authorities to carry out supervision over. Thus, the size of the investment opportunities can lead to the complexity of reporting company business transaction information, leading to the tax authorities' inability to supervise. The lack of optimal supervision by the tax authorities over companies results in a low amount of company tax payments, indicating tax avoidance practices. This study's test produced empirical evidence that companies with large investment opportunities tend to avoid taxes.

The investment opportunity set owned by the company describes the tangible assets or resources owned by the company and the company's ability to continue to grow by investing in various investment options that are profitable for the company (Gaver and Gaver, 1993; Kallapur and Trombley, 2001; Myers, 1977). Based on the descriptive statistical analysis results, the manufacturing companies in this study generally have a small or limited set of investment opportunities. However, some manufacturing companies have a large set of investment opportunities. Based on the hypothesis testing results, if a manufacturing company has an investment opportunity set, the investment opportunity set tends to encourage the company to avoid taxes.

The descriptive statistical analysis results suggest that although there are no specific provisions regarding investment opportunity set criteria and reporting mechanisms in Indonesia, several companies engaged in the manufacturing sector have a large set of investment opportunities. During the period 2014–2019, the manufacturing sector had always been the second-largest investment realization (BKPM, 2020). Thus, although it has not been regulated explicitly in Indonesia, the investment opportunity set tends to have become a company tool to disguise tax avoidance activities.

In the context of Indonesia, empirical evidence obtained in previous study is the investment opportunity set to prevent tax avoidance is marked by the high GAAP ETR of the company's (Lubis et al., 2015). GAAP ETR shows the ratio between the company's total tax expense and its profit before tax (Hanlon and Heitzman, 2010). Meanwhile, using the Current ETR as a tax avoidance measure, the empirical evidence obtained is the investment opportunity set's inability to influence tax avoidance (Handayani, 2013). Both empirical evidence employed tax avoidance measures whose value can be explicitly known in financial statements. Meanwhile, using discretionary accruals to measure tax avoidance, the resulting empirical evidence is the investment opportunity set's ability to reduce tax avoidance (Firmansyah and Bayuaji, 2019). As a tax avoidance measure, discretionary accruals cannot be explicitly known in the financial statements because they must look at the residual value of the BTD regression results (Lim, 2011). The residual value is a BTD component that cannot be explained through corporate earnings management (Lim, 2011). Thus, GAAP ETR, Current ETR, and discretionary accruals cannot capture the tendency of companies in Indonesia to avoid taxes, as seen from the investment opportunity set. In studies conducted outside Indonesia, the empirical evidence obtained regarding investment opportunity sets on tax avoidance is similar to that obtained from previous studies in Indonesia. Using the presence or absence of investment in tax shelter activities or tax savings as an indication of tax avoidance, the investment opportunity set turns out to cause companies in the United States to be less likely to avoid taxes (McGuire et al., 2014). Although using different tax avoidance measures, Indonesia and the United States have similarities in implementing a self-assessment tax system that gives taxpayers the confidence to calculate, pay, and report their taxes (Djulianto, 2015). This study produces empirical evidence that is different from previous studies that have been described above, both in Indonesia and outside Indonesia. By using the abnormal permanent difference originating from the discretion of company managers (DTAX) as a measure of tax avoidance, this study has proven empirically that the investment opportunity set is used as a means of avoiding taxes by manufacturing companies in Indonesia, which is a country with a self-assessment tax system. Therefore, to be able to detect tax avoidance activities in a manufacturing company that has a set of investment opportunities and is in a country with a self-assessment tax system, the measure of tax avoidance must be seen from the abnormal permanent difference that comes from the discretion of company managers (DTAX).

### 4.3 The role of corporate social responsibility disclosure in the association between political connection and tax avoidance

According to the hypothesis testing results, corporate social responsibility disclosure can weaken the positive influence of political connections on tax avoidance. This study's empirical evidence confirms the company's objectives in carrying out corporate social responsibility activities based on stakeholder theory views. According to stakeholder theory, companies are responsible to owners or shareholders and other parties who can influence their sustainability, such as society and government (Freeman, 2010). The company realizes responsibility to the community by implementing corporate social responsibility activities, corporate moral obligation (Freeman, 2010), and corporate social responsibility disclosure, a medium for corporate communication with stakeholders (McWilliams and Siegel, 2001). On the other hand, companies do the same thing to the government by properly fulfilling tax obligations. Corporate social responsibility disclosure can align interests and meet the community and government's demands, including company stakeholders. The harmony between corporate social responsibility and taxes is also shown in this study by the disclosure of economic aspects, which is the disclosure of the value distributed to the government in taxes. Thus, based on this study's results, corporate social responsibility disclosure in line with efforts to reduce tax avoidance practices confirms the stakeholder theory's viewpoint. The results of descriptive statistical analysis in this study have provided information that although there has been no statutory regulation or accounting standard that specifically serves as a legal protection regarding the obligation to implement and disclose social responsibility for all public companies in Indonesia until 2019, public companies in the manufacturing sector in Indonesia.

Generally, public companies have made the disclosures either through sustainability reports or annual reports. Manufacturing companies that have disclosed social responsibility are companies whose business activities are closely related to the management of natural resources and the environment because they carry out the mandate of the Law Number 40 of 2007 concerning Limited Liability Companies and Regulation of the Government of the Republic of Indonesia Number 47 of 2012 concerning Social and Environmental Responsibility of Limited Liability Companies. However, the implementation and disclosure of new corporate social responsibility are required for public companies starting from 2020 based on the Regulation of the Financial Services Authority Number 51/POJK.03/2017 concerning the Implementation of Sustainable Finance for Financial Service Institutions, Issuers, and Public Companies. The level of disclosure of corporate social responsibility is seen from the delivery of information related to company activities that have economic, environmental, and social impacts to meet the information needs of stakeholders that affect the sustainability of the company (Adams and McNicholas, 2007; Salomone and Galluccio, 2001). It also shows the implementation of corporate social responsibility activities. Under descriptive statistical analysis, manufacturing companies in Indonesia generally have implemented corporate social responsibility disclosures. On average, manufacturing companies in Indonesia have a low corporate social responsibility disclosure level. However, based on the results of hypothesis testing, if the company has carried out corporate social responsibility activities even though it is still low, the positive influence of political connections on tax avoidance can be reduced.

This study has proven that Indonesia's manufacturing companies with political connections tend not to avoid taxes when the company has carried out corporate social responsibility activities. Implementing corporate social responsibility activities is not a formality for the company but has made it fulfill its moral obligations properly while meeting stakeholders' demands. The company can still fulfill the community and government's interests as company stakeholders by carrying out corporate social responsibility activities even though it has political connections. The company can contribute to society in the economic, environmental, and social fields. Besides, the company also contributes to the government by adequately carrying out its tax obligations to achieve the tax revenue target. The implementation of moral obligations and the fulfillment of the demands of corporate stakeholders can still be improved after implementing the obligation to disclose corporate social responsibility to all public companies in Indonesia starting in 2020 (Otoritas Jasa Keuangan, 2017b). With the empirical evidence obtained in this study, the things that underlie the ownership of political connections in manufacturing companies in Indonesia that have carried out corporate social responsibility activities can be presumed no longer for tax avoidance but for other purposes such as increasing the chances of obtaining loans (Firth et al., 2009). Based on the stakeholder theory framework, these two things are carried out by companies to align the interests of stakeholders, including shareholders, society, and the government. With extensive opportunities to get loans and special access to markets, companies can continue growing and generating profits. It makes the company meet stakeholder demands, increase shareholder wealth, increase corporate social responsibility activities to the society, and increase tax paid to the government.

### 4.4 The role of corporate social responsibility disclosure in the association between investment opportunity sets and tax avoidance

Based on the hypothesis testing results, corporate social responsibility disclosure can weaken the positive influence of the company's investment opportunity set on tax avoidance. It shows that the magnitude of the positive influence of the investment opportunity set on corporate tax avoidance can be reduced by disclosing corporate social responsibility in the sustainability report or annual report. This study produced empirical evidence that confirms stakeholder theory. Companies carry out corporate social responsibility activities as a form of moral obligation (Freeman, 2010) and disclose corporate social responsibility as a communication medium with stakeholders, including the community and government (McWilliams and Siegel, 2001). For the community, the company realizes this through implementing corporate social responsibility activities. Meanwhile, companies manifest this in the proper implementation of tax obligations to the government. The harmony between the disclosure of corporate social responsibility with aspects of corporate taxation has also been proven in this study through the high level of disclosure of economic aspects, one of which is the disclosure of the value distributed to the government in the form of taxes.

This research produced descriptive statistics that showed that public companies in the manufacturing sector in Indonesia generally have carried out corporate social responsibility activities and made disclosure of this information through sustainability reports or company annual reports. In fact, up to 2019, there have been no laws and regulations or accounting standards that specifically impose obligations on all public companies in Indonesia to carry out and disclose corporate social responsibility activities. In general, manufacturing companies in Indonesia that have carried out and disclosed corporate social responsibility activities have a close relationship with business activities that manage natural resources and the environment. The level of disclosure of corporate social responsibility is seen from the delivery of information about company activities that have economic, environmental, and social impacts to meet the information needs of stakeholders that affect the company's survival (Adams and McNicholas, 2007; Deegan and Rankin, 1996; Salomone and Galluccio, 2001). It also showed the implementation of corporate social responsibility activities. The descriptive statistical analysis results showed that public companies in Indonesia's manufacturing sector had a low corporate social responsibility disclosure level. However, based on the results of hypothesis testing, if the company has carried out corporate social responsibility activities even though it is still low, the positive effect of the investment opportunity set on tax avoidance can be reduced. This study has provided empirical evidence that manufacturing companies in Indonesia with investment opportunities tend not to avoid taxes when the company has adequately implemented corporate social responsibility activities. Implementing corporate social responsibility activities should indicate that the company has fulfilled its moral obligations properly while meeting stakeholders' demands. Thus, the community and government's interests as company stakeholders can still be fulfilled and harmonized by companies with a set of investment opportunities.

Given the empirical evidence obtained in this study, the things that underlie the ownership of a large set of investment opportunities in public companies in the manufacturing sector in Indonesia that have carried out corporate social responsibility activities can be presumed to be not aimed at avoiding taxes but at other purposes such as getting a positive response from the market and potential investors (Vogt, 1997). A positive response from the market and potential investors can increase the company's investment to continue to grow and increase profits. Besides, manufacturing companies in Indonesia that have carried out corporate social responsibility activities can reaffirm the primary use of the investment opportunity set that the company has, which is as an indicator of company growth (Adam and Goyal, 2008; Gaver and Gaver, 1993) and the company's business strategy (McGuire et al., 2014). Tax avoidance as one of the company's strategies to earn profits is not the primary choice when corporate social responsibility is applied to companies with investment opportunities.

## 5 Conclusions

The strength of the political connections of manufacturing companies in Indonesia as one of the developing countries is seen in the number of directors/commissioners/shareholders with a minimum ownership level of 10% who have held or are currently holding positions in parliament, cabinet, political parties, central/regional government, or military forces, and it can influence the formulation of company policies related to tax avoidance. The set of investment opportunities of manufacturing companies in Indonesia, which are the company's tangible assets and the company's ability to develop through investment, derives from company policies that tend to avoid taxes and cause complexity in the company's business transaction information, which results in less than optimal supervision by the tax authorities so that companies can more easily avoid taxes. The tendency of companies to use political connections to avoid taxes can be reduced by implementing corporate social responsibility, which is a form of corporate moral obligation and the fulfillment of the demands of stakeholders. Manufacturing companies in Indonesia with political connections tend to not avoid taxes when they have carried out corporate social responsibility. Companies' tendency to use the investment opportunity set as a tax avoidance tool can be reduced by implementing corporate social responsibility, fulfilling moral obligation, and meeting stakeholders' demands. Manufacturing companies in Indonesia with a set of investment opportunities tend to not avoid taxes when they have carried out corporate social responsibility.

Based on the discussion that has been presented in the previous section, this study has several limitations. The exclusion of manufacturing companies with negative income before tax resulted in a significant reduction in the sample of this study by 54 companies, thus reducing the number of samples. Therefore, this study's results can only describe public companies' conditions in Indonesia's manufacturing sector but cannot generalize all public companies in Indonesia. This study's index score for corporate social responsibility disclosure employed the content analysis method. The use of content analysis methods in a study cannot be separated from researchers' subjectivity. Future studies can use the object of a public company engaged in a sector other than manufacturing to explain companies' research variables' nature in other sectors.

Further studies can also use all company sectors on the Indonesia Stock Exchange to know the effect of research variables on all companies and analyze each sector in-depth. Besides, future studies can analyze public companies in several countries, both those that adhere to and those that do not adopt the self-assessment tax system, to determine the influence of research variables based on each country's characteristics and the tax system applied. Further studies can use a more extended analysis time range to capture the influence of political connections and investment opportunity sets on tax avoidance and the role of corporate social responsibility disclosure in moderating the effect of these two variables on tax avoidance. Future studies can also add the company's political connections based on company contributions to political party campaigns while holding general elections.

This study indicates that the Indonesian Tax Authority should focus on sustainability issues in refining existing tax policies. It can also collaborate with parties authorized to issue policies and regulations regarding implementing and disclosing corporate social responsibility. With the existence of good synergy among regulators, it is expected that a conducive economic climate can be created, which will lead to the optimization of tax revenues. More technically, the Authority can also map corporate taxpayer profiles based on the characteristics of political connections and the company's investment opportunity set. The results of the taxpayer profile mapping can be the basis for Account Representatives in extracting tax potential. Tax auditors can also conduct tax audits of companies with strong political connections and big investment opportunities. Political connection criteria and investment opportunity sets can also enrich the criteria of taxpayer compliance risk management and specific audit criteria for manufacturing companies to minimize the risk of tax avoidance.

As an institution in charge of regulating and supervising public companies in the Indonesian capital market, the Indonesia Financial Services Authority should issue generally applicable regulations and regulate politicians who have the potential to create political connections to public companies in Indonesia. This regulation is expected to complement or improve the current regulation, which does not explicitly mention political connections and regulates the financial services sector.

Furthermore, this study indicates that prospective investors should consider that companies that conduct tax avoidance have the potential to suffer future losses due to the obligation to pay tax sanctions or penalties due to non-compliance with tax regulations. Thus, potential investors can use information about the political connections and investment opportunity set that the company has to determine the degree of the company's tendency to avoid taxes.

As a state institution mandated to manage investment in Indonesia, regarding this study’s findings, the Indonesia Investment Coordinating Board should establish provisions related to criteria and reporting on the set of investment opportunities owned by companies. These criteria and reporting are expected to generate synergy between public companies and the government. Good synergy can increase company investment and, finally, increase state revenue.

## Declarations

### Author contribution statement

Amrie Firmansyah: Conceived and designed the experiments; Performed the experiments; Analyzed and interpreted the data; Contributed reagents, materials, analysis tools or data; Wrote the paper.

Amardianto Arham: Conceived and designed the experiments; Performed the experiments; Analyzed and interpreted the data; Contributed reagents, materials, analysis tools or data; Wrote the paper.

Resi Ariyasa Qadri: Performed the experiments; Analyzed and interpreted the data; Contributed reagents, materials, analysis tools or data; Wrote the paper.

Puji Wibowo: Conceived and designed the experiments; Contributed reagents, materials, analysis tools or data; Wrote the paper.

Ferry Irawan: Performed the experiments; Analyzed and interpreted the data; Contributed reagents, materials, analysis tools or data; Wrote the paper.

Nur Aisyah Kustiani: Conceived and designed the experiments; Performed the experiments; Wrote the paper.

Suparna Wijaya: Performed the experiments; Analyzed and interpreted the data; Contributed reagents, materials, analysis tools or data; Wrote the paper.

Arifah Fibri Andriani: Conceived and designed the experiments; Performed the experiments; Wrote the paper.

Zef Arfiansyah: Performed the experiments; Analyzed and interpreted the data; Contributed reagents, materials, analysis tools or data; Wrote the paper.

Lestari Kurniawati: Conceived and designed Con Performed the experiments; Analyzed and interpreted the data; Contributed reagents, materials, analysis tools or data; Wrote the paper. ceived and designed the experiments; Performed the experiments; Wrote the paper.

Azas Mabrur: Conceived and designed the experiments; Performed the experiments; Wrote the paper.

Agung Dinarjito: Performed the experiments; Analyzed and interpreted the data; Contributed reagents, materials, analysis tools or data; Wrote the paper.

### Funding statement

This research did not receive any specific grant from funding agencies in the public, commercial, or not-for-profit sectors.

### Data availability statement

Data will be made available on request.

### Declaration of interest’s statement

The authors declare no conflict of interest.

### Additional information

No additional information is available for this paper.
